# Graduates feedback on the master of public health program in the United Arab Emirates: a mixed method study

**DOI:** 10.3389/fpubh.2025.1717098

**Published:** 2026-01-12

**Authors:** Iffat Elbarazi, Ismail Elkonaisi, Luai A. Ahmed, Azhar T. Rahma, Preetha Menon, Marília Silva Paulo, Michal Grivna, Javaid Nauman

**Affiliations:** 1 Institute of Public Health, College of Medicine and Health Sciences, United Arab Emirates University, Al-Ain, United Arab Emirates; 2 Health Education Research Unit, Institute of Public Health, College of Medicine and Health Sciences, United Arab Emirates University, Al-Ain, United Arab Emirates; 3 NOVA National School of Public Health, Public Health Research Centre, Comprehensive Health Research Center, CHRC, REAL, CCAL, NOVA University Lisbon, Lisbon, Portugal; 4 Department of Circulation and Medical Imaging, Faculty of Medicine and Health Sciences, Norwegian University of Science and Technology, Trondheim, Norway; 5 Healthy Living for Pandemic Event Protection (HL-PIVOT) Network, Chicago, IL, United States

**Keywords:** career impact, curriculum development, graduate evaluation, master of public health, post graduate studies, public health education

## Abstract

**Background:**

The increasing complexity of public health challenges necessitates well-trained professionals equipped with a diverse range of competencies. The Master of Public Health (MPH) programs play a crucial role in preparing the workforce to address emerging health issues. This study evaluates the MPH program at the Institute of Public Health, College of Medicine and Health Sciences, United Arab Emirates University (UAEU), to assess its impact on graduates' career progression, competency application, and areas for improvement.

**Methods:**

A mixed-methods approach was employed, using an online survey and in-depth interviews with MPH alumni. A structured questionnaire was distributed to 99 graduates, with a response rate of 25% (*n* = 25). Additionally, semi-structured interviews were conducted with 15 graduates. Quantitative data were analyzed using SPSS, while qualitative data underwent thematic analysis with NVivo software.

**Results:**

The median age of the participants was 38 years, about 80% of the respondents were female, and 88% were employed full-time. Overall, 52% rated the MPH program as excellent, and 64% reported that it met their expectations. However, only 32% experienced career progression, while 60% faced challenges in securing relevant job opportunities. Research engagement remained low, with only 8% publishing in peer-reviewed journals post-graduation. The participants also identified gaps in practical training, methodological instruction, and research opportunities, suggesting the need for a thesis track, structured internships, and expanded coursework in digital health, policy analysis, and leadership.

**Conclusion:**

While the MPH program at UAEU is well-regarded, improvements in curriculum design, practical training, and research support are necessary to enhance its impact on career advancement and public health practice. Addressing these gaps will better align the program with evolving workforce demands.

## 1 Introduction

The COVID-19 pandemic and recent global warming-related disasters highlighted the need for a highly trained health workforce in curative and public health services involving surveillance, health promotion, health communication and logistic systems (1). A resilient and adaptive health system requires an adaptive workforce equipped with the skills and competencies to meet diverse health needs and changing health priorities (1, 2). Therefore, investing in health systems includes investing in the health workforce (3).

In the past, education in epidemic control and statistics was primarily reserved for individuals with medical backgrounds (4). This restriction existed partly because early public health programs were primarily offered by medical schools, typically under the categories of preventive health or community health. Although, the boundaries between these fields were somewhat indistinct, various schools of public health eventually emerged, incorporating a multidisciplinary approach that introduced expertise in administration, statistics, and field programs (4). Over time, many of these programs expanded their faculty to include experts from fields such as economics, sociology, statistics, and behavioral sciences. In recent decades, a series of catastrophic events, with the most recent being the COVID-19 pandemic, have compelled the public health education system to broaden its curriculum pedagogical approaches. Beyond expanding content in areas like supply chain management, health communication, and decision science (5), the pandemic accelerated the adoption of online and blended learning formats across higher education globally. While many institutions had begun implementing blended learning initiatives prior to the pandemic, the emergency shift to fully online instruction in 2020 often occurred without adequate preparation time or prior institutional experience with online pedagogy. This context is relevant for understanding the student feedback as participants' experiences reflect a period of institutional adaptation and learning.

The transformation in public health education has been characterized by a gradual shift toward competency-based curricula, which aims to bridge the workforce skill gap (6). As a result of this shift, various courses were designed and offered within diplomas and master's programs to encompass the diverse competencies essential for public health systems (7, 8). The Master of Public Health (MPH) programs around the world are currently addressing both the knowledge base and workforce competency gap in the field of public health simultaneously (9, 10).

While curricula offered in MPH have been modified to meet the growing health needs of the population, the mode of delivery has also evolved to align with technological advances and the needs of learners (11). The MPH programs have evolved from traditional 1–2 year residential programs to include more contemporary options like part-time, weekend, executive, and online programs that typically span 2–3 years (3, 6). Moreover, the MPH programs expanded to accommodate graduates from non-medical backgrounds like engineering, social science, management and law (4, 9, 11), thus, enriching the talent pool of professionals entering public health practice.

An MPH program serves a role beyond equipping professionals with working knowledge of epidemiology, biostatistics, disease prevention and environmental health (3, 5). Public health research skills gained through the master's program help improve health system efficiency and health system reforms (3).

Evaluation is an essential accountability measure for educational programs (12, 13). Continuous evaluation of master's programs involves evaluating curricula and the mode of teaching to meet the changing needs of health professionals (6). Regular assessment of public health education requirements followed by evaluation of present programs in meeting these requirements (2) is standard practice in most countries. However, measuring the impact of higher education programs like the MPH can be challenging due to various factors like prior graduate education, workplace environment conducive to employing learned skills, and the heterogeneity of public health work profiles (3). This makes student feedback one of the most common tools of evaluation. The MPH students' feedback usually comes from public health professionals who are well-attuned to the needs of the system and their ability or inability to meet them. Despite the lack of a standardized tool, student feedback is the best source to assess the program's impact (14, 15). It helps fill the skill gap and direct reforms, and innovations in public health education. The evaluations measure the content and mode of delivery in their ability to meet the needs of the public health professionals and their organizations (6). Different masters' programs in public health have been evaluated chiefly by student surveys (3). In-depth interviews provide a rich mode of organizational learning and help validate the survey results (16).

In 2019, there were 428 million people living in the 22 Arab League member nations. Some of the richest and poorest nations in the world are represented here. While other nations continue to be plagued by diseases of poverty, many nations experience a high burden of non-communicable diseases and injuries (1). Inequities in health, increased exposure to health risks, rising health care costs, and intolerably low levels of access to high-quality healthcare have been named as the region's main problems. Moreover, insufficient funding for health-related research, the need to reform public health education, and the ongoing difficulties experienced by nations with complex emergencies may be added to this list (2). The MPH programs in this region date to over a 20-year period and were mainly established by the High Institute of Public Health (HIPH) at the University of Alexandria, Egypt, and the Faculty of Health Sciences at the American University of Beirut (AUB), both of which were founded in 1971 (1). Currently the MPH programs are distributed among 13 countries in the Arab regions including Lebanon, Palestine, Oman, Saudi Arabia, Somalia, Sudan, United Arab Emirates (UAE), Egypt, Jordan, Kuwait, Mauritania, Morocco, and Qatar (1). To our knowledge, several universities around the region have initiated MPH programs after the COVID-19 pandemic to meet the demand of their own countries or as part of their institutional strategies, which attract more students, increase funding, and enhance reputation and ranking (17).

**Aim:** Our primary goal was to evaluate the MPH program offered by the Institute of Public Health (IPH) at the College of Medicine and Health Sciences (CMHS) of the United Arab Emirates University (UAEU). We adopted a mixed-method approach, with a central focus on gathering students' feedback for assessment.

## 2 Objectives:

1. To analyze the impact of the IPH—MPH program on graduates' career progression and leadership development.
2. To examine how the competencies acquired through the program are applied to meet the evolving requirements of the public health job market.
3. To investigate areas where enhancements can be made to the educational methods and the public health program curriculum to improve the overall learning experience.

## 3 An overview of the IPH—MPH program

The professional MPH at the IPH was the first program initiated in the UAE in 2010. It was established following the influence of the American public health education model (1). The program aims to provide UAEU students with the foundational skills, education, and expertise necessary to work in a variety of public health employment settings including public health departments, non-profit agencies, hospital population health departments/offices and research. The program is oriented toward applied practice and research in public health, and is designed for medical, health and science professionals working in these fields who intend to lead health organizations in the public and private health sectors and in teaching and research organizations. This part-time master's degree course was structured to run from Thursday to Sunday from 09:00 to 17:00 h during the study period. The program required students to accumulate 34 credits over a minimum of 3 years (six semesters). During the first year, students typically enrolled in three courses per semester, each worth two credits. From the second year onwards, students could register for one research assignment (two credits) per semester. All participants in the present study graduated prior to 2021 and experienced this structure throughout their enrollment. The program structure has since been modified to incorporate blended learning, combining in-person instruction with online modules through a flipped classroom approach.

The program comprises 28 credits from courses and six credits from research assignments distributed across three sequential Public Health Assignment courses: Introduction to Scientific Writing (Assignment I), Critical Literature Review (Assignment II), and Research Protocol or Program Planning (Assignment III), designed to develop students' research, analytical, and scientific writing competencies, with the expectation that at least one assignment reaches publishable quality under faculty supervision. Students are expected to spend time on self-directed study and homework assessments. For every hour that is spent in the classroom, students should spend 2 h on self-directed study. Students select from 14 courses covering public health, epidemiology, biostatistics, health promotion, public health management, health protection, environmental and occupational health, maternal and child health, and global health.

The MPH program follows a spiral curriculum advanced by Bruner (1960). As such, course content and the sequence of courses are structured so that complex ideas are covered at simplified levels first and then re-visited at more complex levels later in the program. Topics are taught at levels of gradually increasing difficulty (hence, the spiral analogy). Ideally, teaching this way should lead to students becoming independent learners, critical thinkers and effective practitioners in public health. The program was accredited by the Western Association of Schools and Colleges (WASC) in 2015, and by the UAE Commission of Academic Accreditation (CAA) in 2017. A reaccreditation was conducted this year, 2025. To date, the program has graduated more than 100 students. In 2018, an alumni group was created to help the IPH faculty and staff to track the successes of the program graduates with the aim to improve the program to meet the UAE market needs in public health practice. Alumni were invited to be part of the IPH activities including the IPH quarterly newsletter and to help in teaching and research when possible.

Since its establishment, the MPH program at the IPH has improved on course topics, and various educational models were introduced through teamwork and individual efforts; however, a formal assessment and evaluation of the program outcomes and impact on the public health practice has never been conducted. With the emergence of the COVID-19 pandemic, the IPH has been receiving requests from federal and local public health departments to provide workshops and training for health care workers in the areas of public health including epidemiology, biostatistics, health promotion and evidence-based interventions. These requests in addition to the actual global situation that the COVID-19 created have uncovered significant gaps in appropriate public health policies and strategies and unveiled the substantial gap in training and equipping public health experts and professionals to face such challenges. This local situation in the UAE and surrounding countries is not different from the global situation calling for immediate action to meet the market needs of skilled and professionally trained public health specialists (18). The IPH being one of the leading institutes and one of the earliest departments to develop public health programs decided to take immediate action starting with an evaluation and assessment of the actual public health program offered at the UAEU using the Kirkpatrick framework of evaluation training programs (19).

## 4 Methods

This is a mixed-method study that utilizes both quantitative and qualitative approaches. To collect the quantitative data, an English language questionnaire was created based on literature review, previous evaluation studies, and input from public health professionals (20, 21). The research team carefully examined the questionnaire for language and content, and it was then pre-tested with former students who had worked as research assistants at the IPH, where the study was conducted. There was no need to validate the questionnaire as this is more an exploratory study.

In addition, an interview guide was constructed based on the questionnaire to be used in face-to-face interviews with alumni of the MPH program. The interview guide was created by an experienced qualitative researcher and was reviewed by experts from the IPH faculty members who had previous experience in coordinating and teaching on the MPH program. Subsequently, the interview guide was piloted with two enrolled MPH students, and a PhD candidate who has an MPH from another institute. Relevant questions and probes were added to ensure the comprehensiveness of the interview guide which was constructed and administered in English language.

**Ethics:** The study was approved by the UAEU Social Science Ethics Committee- Approval number ERS-2021-7299.

**Inclusion criteria:** Graduates from the IPH-MPH program who graduated between the year 2012 and 2020 and who accepted to be part of the study. Those who were still enrolled in the MPH program were excluded.

**Institutional context: program evolution and COVID-19 pandemic transition:** Prior to the COVID-19 pandemic, the UAEU had initiated institutional efforts to support blended learning through the Center for Excellence in Teaching and Learning (CETL), and faculty had received exposure to blended learning concepts. However, the MPH program remained primarily face-to-face, with Blackboard used mainly for administrative functions rather than active blended instruction. In March 2020, the pandemic necessitated an abrupt shift to online instruction utilizing Blackboard Ultra, Zoom, and Microsoft Teams. While institutional support including faculty training was provided, the rapid transition allowed limited preparation time for curriculum redesign or comprehensive faculty development.

The participants in our study graduated between 2012 and 2020, meaning their experiences ranged from exclusively face-to-face instruction (pre-March 2020) to online instruction during the pandemic transition. This variation is important for interpreting student feedback regarding online learning, as it reflects experiences during institutional adaptation rather than deliberate pedagogical design. Since study completion, the program has implemented blended learning as a core approach with both in-person and online components.

**Sampling:** A convenience purposive sampling was used to recruit participants for the survey. The alumni list was obtained from the UAEU graduate office administrator, and verified by the MPH coordinators and the postgraduate office at CMHS. At the time of the study (conducted between June 2021 and June 2022), a total of 99 MPH students had graduated. All alumni members were contacted via their personal and institutional emails. All participants received an invitation email that included an information sheet about the study, consent form, and a link for the survey. A follow up on WhatsApp was sent to those whose numbers were available. Those graduates who we still had contact with were called via the phone to invite them to participate. Another email invitation was sent to participate in the face-to-face interviews. Given the initial low response rate, we sent periodic reminders, and used snowball techniques to maximize recruitment.

### 4.1 The questionnaire and the interview guide

The online questionnaire was divided into three main categories: questions related to demographics, questions on work experience and impact of MPH on career and research as well as personal experience during the enrollment in the program. The interview guide was also divided into three parts (personal characteristics, current work and change in career as well as personal views about the program). Probes and prompts as well as suggestions were recorded. All interviews were audio recorded after obtaining consents from participants.

### 4.2 Statistical analysis

Quantitative data from the questionnaire was analyzed using IBM SPSS software (version 29). Descriptive statistics are presented including the participants' review of the MPH teaching, course content and instructional methods. The qualitative data was analyzed using Braun and Clarke's (2006) thematic analysis using NVivo software (22). Thematic analysis is a qualitative research methodology that involves systematically identifying, analyzing, and reporting patterns (themes) across qualitative data to develop a rich understanding of the research phenomenon. Both inductive and deductive coding approaches were used. Inductive coding involved generating codes directly from the data without predetermined categories, while deductive coding applied *a priori* themes relevant to the study objectives. This dual approach enabled the identification of both expected and emergent themes related to program strengths, challenges, and areas for improvement. All interviews were recorded after obtaining participants written consent and then transcribed into verbatim text. Two experts in qualitative research methodology reviewed the transcripts and coded the data to identify initial themes and sub-themes. Then they sat together with a senior expert in qualitative research and verified, compared, and reconciled coding discrepancies to ensure consistency and validity. Through iterative discussion, the three researchers reached consensus on the final set of themes and subthemes. Thematic saturation was assessed and reached by the 10th interview, suggesting that no new themes emerged from subsequent interviews. However, as we were conducting the study using convenience sampling, all individuals who responded to interview invitations were included in the analysis, resulting in 15 completed interviews.

## 5 Results

### 5.1 Questionnaire

**Figure 1:**
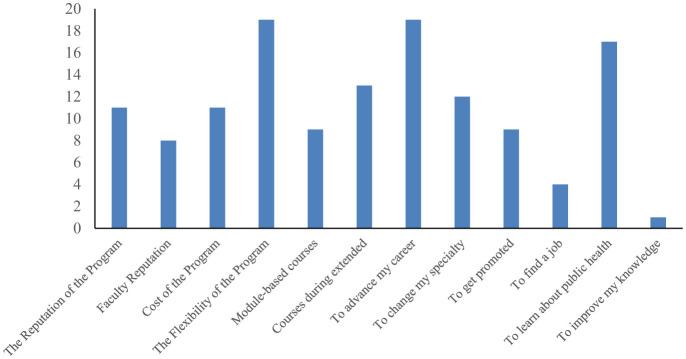
Reasons to join the MPH program.

Only 25 out of 99 graduates responded by filling in the survey, making the response rate only 25%. The median age of participants was 38 years. Five were males, and eight were Emirati national. A majority of the participants were full time employees (*n* = 22), eight were nurses, five medical doctors, two academics, and the rest were other allied health care professionals (Table 1). Most reasons for joining the program included the interest in public health discipline, flexibility and reputation of the program, and to advance careers (Figure 1).

**Table 1:** Participants' demographics, employment status, and program characteristics.

**Characteristic**	***n =* 25**
**Age (median, IQR), years**	38 (33.5–40.5)
**Female gender**	20 (80%)
**Nationality**	
UAE	8 (36%)
Others	17 (64%)
**UAE resident**	
Yes	20 (80%)
No	5 (5%)
**Emirate of residency**	
Al Ain and Abu Dhabi	21 (84%)
Other Emirates	4 (16%)
**Marital status**	
Married	17 (68%)
Single	8 (32%)
**Number of children**	
0	7 (28%)
≥1	18 (72%)
**Current employment status**	
Full-time	22 (88%)
Part-time	1 (4%)
Unemployed	2 (8%)
**Professional background**	
Nurse	8 (32%)
Physicians	5 (20%)
Allied	5 (20%)
Academic (research/lecturer)	2 (8%)
**Prior postgraduate education**	
Had other postgraduate degrees	11 (44)
This was only postgraduate degree	14 (56)
**Graduation period**	
2010–2014	6 (24%)
2015–2018	14 (56%)
2019–2020	5 (20%)
**Delays/interruptions during studies**	
No delays	24 (96%)
Reported delays	1 (4%)
**Preferred program track**	
Thesis-based	15 (60%)
Non-thesis	10 (40%)
**Curriculum choices**	
Extended weekends	18 (72%)
Online or evening classes	4 (16%)
Day classes	3 (12%)

Data are number (%), otherwise indicated.

IQR, Interquartile Range.

Professional background categories based on self-reported current/most recent professional role at time of study. Graduation period reflects cohorts: Early (2010–2014), Mid (2015–2018), Recent (2019–2020). Preferred track based on interview data indicating which program structure (thesis vs. course-based) respondents felt would be most valuable.

We found that 96% (24 students) did not experience any delays or interruptions during their MPH studies. Only one student reported a delay, which was attributed to health issues. There were no reported delays due to lost interest, administrative issues, financial constraints related to program tuition or fees, conflicts with the college or faculty, or traveling. Most students reported that this was their only postgraduate degree while 11 had other postgraduate degrees (Table 1). Some reported (32%) that they got employment after graduation while more than half (52%) remained in the same position (Table 2).

**Table 2:** Post-graduate career outcomes and research engagement.

**Variable**	*n =* 25^*^
**Employment post-graduation**	
Obtained new employment	8 (32)
Changed jobs within 1 year	4 (16)
Remained in same position	13 (52)
**Career progression**	
Experienced promotion/advancement	8 (32)
No career advancement	17 (68)
**Job opportunities in public health field**	
Found relevant opportunities easily	10 (40)
Faced challenges securing relevant positions	15 (60)
**Research engagement post-graduation**	
Participated in research activities	12 (48)
Published as co-author in peer-reviewed journals	2 (8)
Never published	23 (92)
**Barriers to research/publishing**	
Time constraints	18 (72)
Lack of encouragement	7 (28)
Lack of funding/research team	6 (24)
**Skills application in current role**	
MPH competencies applicable to current work	14 (56)
Limited application of MPH training	11 (44)

Data are number (%), otherwise indicated. ^*^For some variables, percentages are based on the total sample, and are not mutually exclusive.

Employment post-graduation: Multiple statuses could apply within one-year post-graduation.

Career Progression: Defined as promotion, title change, salary increase, or expanded responsibilities attributed to MPH degree.

Job Opportunities: Based on respondent perception of ease/difficulty finding positions aligned with MPH preparation.

Research Engagement: Low publication rate (8%) reflects known barriers: part-time student status, full-time employment (88%), limited institutional research support.

Skills Application: Respondents assessed relevance of learned competencies (epidemiology, biostatistics, health promotion, etc.) to their current roles.

**Figure 2:**
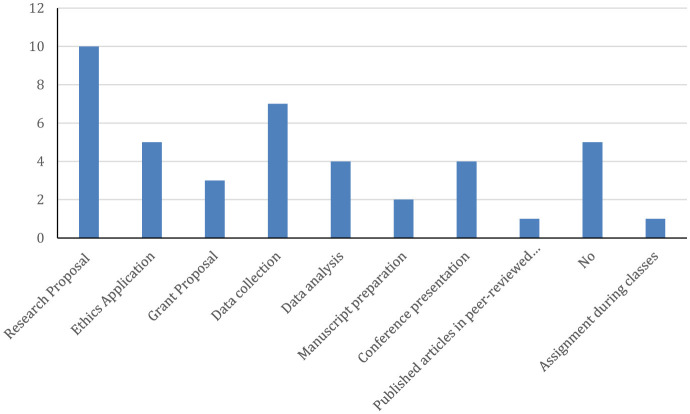
Participating in research while studying the MPH.

Some students reported that they participated in research activities with only two reported publishing as a co-author (Figure 2; Table 2) stating the most common reasons for not publishing were time constraints followed by lack of encouragement, lack of funding and team (Table 2). The MPH contributed to the educational progress of the participants, providing them with more opportunities for building their scientific careers, such as writing scientific papers. All participants provided positive feedback about the MPH program with 52% rating it to be excellent and meeting expectations (Table 3). Only one participant reported a neutral response to meeting expectations while eight reported that the program partially met their expectations, and a total of 10 claimed that the program had gaps. Only one reported that the program did not improve their opportunities while eight reported that the program provided them with career options (Table 3). When participants were asked about areas of improvements in the program, the most commonly cited areas were practical experience, addition of thesis track option, and assignment format and style. Other areas mentioned less frequently (12–20%, *n* = 3–5 each) included course structure and organization, specific course topics, class timing and scheduling, teaching format and delivery methods, and journal clubs (Table 4).

**Table 3:** Program satisfaction, expectations, and overall perception.

**Variable**	*n =* 25^*^
**Overall program rating**	
Excellent	13 (52)
Very Good	9 (36)
Good	3 (12)
**Program met expectations**	
Fully met expectations	16 (64)
Partially met expectations	8 (32)
Neutral	1 (4)
**Program improved career opportunities**	
Provided new career options	8 (32)
Enhanced current position	14 (56)
Did not improve opportunities	1 (4)
Uncertain impact	2 (8)
**Identified program gaps**	
Reported significant gaps	10 (40)
No notable gaps	15 (60)
**Faculty quality and support**	
Highly satisfied with instructor expertise	22 (88)
Satisfied with faculty flexibility/support	20 (80)
Satisfied with instructional methods	19 (76)
**Program value overall**	
Recommend to colleagues	18 (72)
Would pursue again if given choice	21 (84)

Data are number (%), otherwise indicated. ^*^For some variables, percentages are based on the total sample, and are not mutually exclusive.

Overall Rating: Based on direct question: “How would you rate the overall MPH program?” using 4-point scale.

Met Expectations: Based on respondent's self-assessment of whether program content, instruction, and outcomes matched pre-enrolment expectations.

Career Opportunities: Respondents assessed whether MPH enhanced, maintained, or limited their career prospects post-graduation.

Faculty Quality: Satisfaction with expertise, accessibility, understanding, and support; 88% reported highly satisfied with instructor knowledge.

Program Value: Composite of satisfaction metrics; 84% would pursue the degree again despite identified gaps.

**Table 4:** Areas for program improvement identified by graduates.

**Variable**	*n =* 25^*^
**Curriculum and content**	
Expanded practical training/internships	12 (48)
Addition of thesis track option	11 (44)
More biostatistics instruction	8 (32)
SPSS software training	7 (28)
Additional courses (qualitative research, infectious diseases, occupational health, social determinants)	6 (24)
**Instructional methods and format**	
More hands-on/experiential learning	10 (40)
Case study discussions	5 (20)
Journal club activities	4 (16)
Improved assignment timeline/less time pressure	5 (20)
**Program structure and scheduling**	
Adjusted class timing for better accessibility	4 (16)
Continuous engagement between courses	3 (12)
Hybrid/flexible learning options	3 (12)
**Research and publication support**	
Publication requirements/support	6 (24)
Research methodology instruction	5 (20)
Post-graduation alumni research network	3 (12)
**Learning resources**	
Additional reading materials	5 (20)
Extended access to course materials post-graduation	4 (16)

Data are number (%), otherwise indicated.

^*^For some variables, only available data is presented; percentages are based on the total sample, and are not mutually exclusive.

### 5.2 Interview results

Among those who responded to the survey questionnaire, 15 accepted to join an online or face-to-face interviews to further discuss the MPH program, and to provide feedback. Each interview lasted around 30–45 min, and was recorded either on MS Teams (online), or on a digital recorder (face-to-face). All interviews were conducted in English, after obtaining consent. The recordings were transcribed verbatim, and were analyzed by two experienced qualitative researchers (IE and PK).

We identified six major themes and six subthemes. Major themes included: “Program Structure”, “Experience and Influence”, “Learning Experiences and Curriculum”, “Expectations”, Gaps and Challenges”, and “Suggestions”. The subthemes included: “Instructional Method”, “Time and Schedule”, “Program Tracks and Mode”, “Influence on Knowledge” and on “Career”, and “Met and Unmet Expectations”.

### 5.3 Theme one: program structure

The study identified several sub-themes within major theme of program structure, including mode of delivery, program track (thesis vs. non-thesis), program's time and scheduling.

*Mode of Delivery:* The MPH program was originally designed for in-person teaching, but the COVID-19 pandemic necessitated a complete shift to online learning. Students generally expressed a preference for attending classes in person, as it enhanced their understanding of the material and their connection to their academic environment and peers. For example, M2_2019 stated, “*I prefer the attendance in the class... it helps you absorb the course much better compared to online”. Program Track and Mode:* The findings highlighted the importance of program delivery mode, a preference for thesis-based programs, the impact of the shift to online learning due to external factors like the pandemic, and the popularity of part-time scheduling among employed students for its flexibility. Moreover, non-thesis-based programs were less favored by students, who believed that thesis-based programs offered more post-graduation opportunities. They recognized the value of the skills acquired through thesis work, such as research structuring and writing. F11_2014 emphasized this, stating, “*Course based is really enjoyable, but what we learned during thesis... like structuring your thesis... There are certain skills which are very important for the future, especially those who would like to have their research to be written. So, uh, I'd like to see our MPH with thesis... dissertation, yes”. Time and Schedule:* During discussions about part-time programs, it became evident that part-time learning was the preferred option for employed students. This flexibility allowed them to balance their class attendance with their professional responsibilities. F14_2015, now a PhD student, was once a master's student in the MPH program and noted, “*For me, when I was in a master program, I was also like what I am now, a PhD student; I was part-time. I was studying and working. So, the four days per month for each course allowed me to manage it between work and university”*.

### 5.4 Theme two: experiences and influence

Sub-themes identified under this major theme included influence on knowledge and skills, influence on personality, and influence on career opportunities. The participants pointed out that one of the main outcomes of implementing the master program in public health is to develop a better perception and knowledge of public health in the community of UAE. They reported gaining skills such as writing research and proposal, designing prevention programs, finding evidence-based information, and referencing, medication management, having different perspective to view disease, illness, and treatment, understanding how to conduct critical appraisal, and conducting research. Overall participants reported that they gained new concepts in public health.

F9_2017 commented that the master program improved her knowledge in designs of public health studies. “*During my master, I get a benefit and it to enrich my knowledge about the type of the designs of studies”*.

F5_2018 also commented that her writing skills improved well during the program.

“*My writing skills become better this because they always asking about assignments. “PH gave me different tools to educate the community”*.

Participants also reported that the MPH course improved their career opportunities and were able to get promotion while few reported that their MPH was not taken into consideration. Also, some participants reported that the MPH courses can be applicable in their work and within their professional life.

F3_2016: “*It gave me different perspective to view disease, illness and treatment and new concept and approach about wellness”*.

Another important subtheme was the influence on promotion, career change/shifting, applying of MPH courses in career, opening new doors in career, and fulfill gaps of health system. F1_2018 had benefited from courses in her career and commented the following:

“*I got it because I'm working on public health institution, so I felt like now I have some concepts in my mind and some information related to our research that we are doing now”*.

M7_2017 reported that MPH program had significant impact in his career, he directly got a new position in his job after his graduation, and stated: “*I was working as an assistant in school health and university program, when I graduated, they directly put me in charge of the head of department”*.

### 5.5 Theme three: learning experiences and curriculum

The MPH curriculum is based on presentations, lectures, assessments, and post course assignments. The sub-themes extracted were lectures and presentation, course work, discussion, and debates. Overall, the students enjoyed the lectures and course work. Some of the students were satisfied with the curriculum and the assessment methods. Although there were some comments on the assignments and assessments that will be discussed in the gaps section.

F2_2018 and M2_2019 answered sequentially the following: “*I like the diverse methods on work group, working as a group and the discussions and the assessments that we have to do at the end of the course”. And “of course, the lectures were important. The group discussions are important also”*.

The participants mentioned a lot of the important topics they learned during the course and reported different educational and instructional methods that have improved their learning and the education process such as debates, presentations skills, writing skills and public speaking. So many reported enjoying the coursework, discussion and the assignments. They reported being highly satisfied with faculty members backgrounds and experiences being “Knowledgeable instructors” and based on that few students reported recommending the course to other colleagues and friends. One of the very important findings that many students reported was their satisfaction with the program due to its flexibility of the course time and schedule as well as flexibility of the faculty members and their understanding.

### 5.6 Theme four: expectations

The sub-themes include the met and unmet expectations of the students with the program. The met expectations included improving their research skills, improving solutions for health issues, improving prevention programs, improving academic writing and developing career and experience.

F3_2018 had a chance to change her career after obtaining her master's degree on MPH and she said: “*I was planning to change my career and from the first tier and after finishing the first tier of master's degree I got an interview with the in the Dubai Health Authority”*.

On the other hand, some participants found that some expected skills were unmet. F4_2018 had an expectation to develop more skills in writing and reading and commented as following: “*So, I think academic writing is definitely something that needs to be stressed on more because that's the only thing where if you can confidently say that oh I'm able to review papers and I'm able to, you know, let's say come up with, you know, just I have so and so publications under me”*.

### 5.7 Theme five: gaps and challenges

Generally, the major challenges reported by participants were traveling to Alain city the location of the UAEU, the online learning and shortage of time, assessment and submitting required tasks on time as sometimes they were given a short time to submit.

F12_2012 said: “*To come up with presentations and of course it was challenging because we need to work on the presentations while attending the course”*.

F3_2018 said: “*Usually there is a task that we have to do on the 4th day, and that was really very stressful because, uh, because of shortage of time”*.

F3_2018 mentioned: “*Well, I remember it was challenging the way that even long running through it was online and also it was in Group work”*.

Some students preferred to be on campus believing in the importance of interacting with other colleagues and faculty members and teachers.

F6_2018 said: “*I would like to be on campus. I don't like the online, you know because before I join the program of master program I was searching for many online programs, and I really was so amazed with the syllabus and I was about to join and but after that I realized that on campus It's much better you will meet with the people you will have connection. It's really something different from online and for us. Unfortunately, my luck was not so good because for me it comes with the COVID. So, I got part of it as online”*.

On the other hand, some students who are living outside Al-Ain found it difficult to travel during the courses, and COVID-19 brought a better alternative for them to stay in their city without facing the difficulties in traveling.

F3_2018 shared her experience and said: “*My main challenge was traveling to Al Ain because I'm Staying in Sharjah, this was one of the challenging, but it was also Subhanallah (Glory to God)... and when Corona came It helped me that this online learning it was something that I benefited from. Traveling was an issue and then the courses themselves, I enjoyed most of them”*.

Significant gaps were listed by students including missing some required courses and some of the topics not being taught in depth such as occupational health, infectious diseases. Some students expressed their dissatisfaction due to the Insufficient training in SPSS, Insufficient classes in biostatistics, lack connection with the university after courses, some teaching methods, and lack of collaboration with other entities.

F12_2012 stressed on that some wanted courses were unavailable and she quoted: “*I felt that genomics public health genomics. Wasn't there. Population health wasn't there, and the qualitative part wasn't there. You know you had. You had to teach me a lot in the qualitative so I think these are the things that need to be there. Social determinant, behavioral science. All these things I think can be added to the program to make it the best messaging please”*.

The long period between the courses created also another type of gap because it makes the students unconnected with the university after the courses.

F11_2014 commented on that by saying: “*The gap between the courses it created some sort of not continuous and then the flow of the information or the flow of the practice was not continuous”*.

One of the major gaps identified by students is the lack of training, internship and placement. Many of the students described the program being perfect if it includes the option of a thesis and if it provides more hands-on experiences. Further details are provided in the Supplementary material (Supplementary Table S1).

### 5.8 Theme six: suggestions and recommendations

The main recommendations and suggestions were related to having a thesis-based program, extending the courses timeline, providing training, having a post reading material and publishing a research paper.

F10_2018, suggested that it's good to have both a practical and a research side during MPH. “*Again, I wish there was like a practical side or thesis. if there are defense something around that. Something likes to publish”*.

M8_2010 shared recommend also that each student should come up with a paper by saying: “*I would highly recommend that everyone should end up with a paper published somewhere”*.

F4_2018 and F9_2017 highly recommended that having an internship will enrich the program as well and they commented respectively. “*I personally feel like that would really add so much value to the whole program Like it would just. Just be like the cream, you know. Because all that it's kind of like you know how I did like I did five years of medical school and there was so much that I learned. But you know, when it comes to practical application you get so much from that one year of experience”*.

“*To have internship, to have a training or to have like to at least to be invited to a conference It was all sort of searching”*.

Moreover, students were keen to receive more reading supplements to keep them in touch with the courses. Further details are provided in Supplementary material (Supplementary Table S1). For example, F4_2018 noted:

“*So perhaps maybe more assignments or more exercises, or you know, like even more further reading material. To keep me in touch because, see what happens is once you graduate from the program, and if you're not in the same field, then you know it's just you become rusty and you know you may you may forget, or you may not be very...you may not pick up where you left off as opposed to when you're already in a class. If you get what I mean”*.

## 6 Discussion

Our results provide important insights into the MPH program at the IPH, UAEU, based on feedback from graduates between 2010 and 2020. Through a mixed method approach, our findings shed lights on the strengths and challenges of the program, particularly in terms of curriculum structure, learning experiences, and career outcomes. From interviews, we extracted six major themes and six subthemes. Under the major theme of program structure, the study uncovered three critical subthemes: mode of delivery, program track (thesis vs. non-thesis), and time and scheduling. These subthemes summarize the preferences and experiences of the MPH graduates.

Participants reported high satisfaction with the curriculum's diverse instructional methods, including lectures, group discussions, debates, and assignments. These methods align with best practices in public health education, which emphasize active learning and interdisciplinary collaboration (23). The positive feedback on faculty expertise further highlights the importance of knowledgeable instructors in fostering a supportive learning environment. The literature suggests that hands-on learning, including community-based projects and internships, is essential for bridging the gap between theory and practice. Recent studies emphasize that as practice-based degrees, MPH programs must ensure that graduates can effectively translate classroom knowledge to real-world public health challenges (24). However, gaps were identified in assignment styles and practical experiences. Practical training is a cornerstone of public health education, enabling students to apply theoretical knowledge to real-world challenges (25–27). The findings suggest a need to enhance these experiential learning opportunities to better prepare students for real-world public health challenges.

While the COVID-19 pandemic necessitated a transition to online education, many participants reported challenges in engagement, interaction, and comprehension. Although online learning has been widely adopted in higher education, many participants expressed a preference for face-to-face instruction, citing better absorption of material and stronger peer connections. This finding aligns with prior research indicating that face-to-face instruction provides opportunities for deeper engagement, social connection, and overall satisfaction among students (28–30). Earlier results also show that online education offers flexibility, particularly for working professionals, its effectiveness depends on multiple factors, including instructional design, interactive elements, and student support mechanisms (31).

However, student feedback regarding online learning challenges should be interpreted within the specific historical context of this study. Participants experienced online instruction primarily during the pandemic emergency transition (beginning March 2020), when the MPH program lacked prior experience with online or blended pedagogy despite institutional blended learning initiatives. While some faculty had received CETL training in blended concepts, the rapid shift to online instruction allowed limited preparation time for curriculum redesign or faculty development. Students who reported challenges with online learning (reduced material absorption, difficulty with peer interaction) reflect difficulties inherent in rapid, unplanned institutional transitions rather than fundamental limitations of online education. Conversely, some participants appreciated online learning for accessibility benefits. The program has since intentionally implemented blended learning with deliberate instructional design, which future evaluation should assess.

Another key discussion point was the distinction between the thesis and non-thesis tracks. Many participants emphasized the value of research training, with a clear preference for the thesis-based option. Previous studies suggest that graduates with a strong foundation in research methodology are better positioned for careers in academia, policy-making, and advanced public health roles (32). However, balancing coursework, research, and professional commitments remains a challenge for many students. Given that 88% of participants were employed full-time while pursuing their degrees, the program's flexible schedule was highly valued. This aligns with adult learning theories, which emphasize the need for educational programs to accommodate working professionals through flexible delivery formats (33).

Despite the appreciation for program flexibility, some students expressed the need for further adjustments to class timings and formats to enhance accessibility. Similar concerns have been raised in other public health programs, where hybrid or modular learning formats have been suggested as potential solutions (34). Given the increasing number of mid-career professionals enrolling in MPH programs, institutions may need to explore innovative delivery models that balance structure with flexibility.

A significant concern among graduates was the limited job opportunities available post-graduation. While most participants enrolled in the MPH program to advance their careers, many found it challenging to secure relevant public health positions upon completion. This finding is consistent with studies highlighting the disconnect between public health education and labor market demands (35). Strengthening partnerships with public health agencies, non-governmental organizations, and healthcare institutions could help address this issue by providing graduates with clearer career pathways. A recent mixed-methods study of MPH alumni similarly found that while most graduates (63.8%) reported improved job performance, only 35% experienced promotion post-graduation, suggesting a broader misalignment between competency development and career progression outcomes (36).

Moreover, while the MPH program enhanced participants' research skills, particularly in areas such as proposal writing and critical appraisal, only a small number of graduates had published in peer-reviewed journals. Time constraints, lack of encouragement, and limited access to research funding were identified as barriers. This is a well-documented issue in academia, where early-career researchers often struggle to balance publishing with other professional responsibilities (37). Targeted interventions, such as structured mentorship programs and dedicated research time, could help address these barriers and support graduates in contributing to the field of public health scholarship.

The demographic breakdown of the participants provides additional insights into the program's reach and impact. Most students were UAE residents, with a significant proportion working in healthcare settings, particularly within SEHA (the Abu Dhabi Healthcare Company). The high representation of nurses (32%) demonstrates the role of MPH programs in enhancing the skills of healthcare professionals who seek career advancement in public health. Previous results suggest that interdisciplinary MPH programs that attract professionals from diverse backgrounds including nursing, medicine, and social sciences—contribute to a more holistic public health workforce (38). However, the findings also reveal that many graduates did not feel that their MPH degree was sufficiently recognized by employers. This reflects broader challenges in the global public health job market, where the value of MPH degrees varies across sectors and regions (39). Strengthening employer engagement and emphasizing the program's alignment with industry needs could improve graduates' job prospects (40, 41).

Around 76% percent of respondents confirmed they wanted the MPH degree to help them advance their careers. The MPH program significantly influenced participants' knowledge, skills, and career trajectories. Additionally, some participants experienced career advancements or shifts into public health roles post-graduation. This has been reported before by others such as Brownson's study which showed improvements in research skills, academic writing, and critical appraisal which are the key competencies in public health practice (35).

The questionnaire-based survey results showed that 60% of the participants found no job opportunities prior to their completion. Fifty percent of the respondents had no job offers. However, the respondents excelled academically in writing research proposals (40 percent of the sample, even though 88 percent had never published a scholarly article in a peer-reviewed journal before). The primary concern is that most respondents (80 percent) published scientific articles unrelated to studies, jeopardizing overall MPH effectiveness in advancing their careers. The COVID-19 pandemic served as a significant driver for the higher interest in MPH because 56 percent of the respondents chose the epidemic outbreak as the main topic they are interested in when participating in MPH. However, not all participants felt their MPH degree was recognized by employers or translated into tangible career benefits. This highlights a broader issue in aligning educational outcomes with labor market demands. Strengthening partnerships with employers and emphasizing transferable skills during the program could enhance graduates' employability (42).

Despite gaining research skills during the program, only two participants published papers in peer-reviewed journals. Time constraints were cited as the primary barrier, followed by lack of encouragement and funding. These challenges are well-documented in academic literature; early-career researchers often struggle to balance publishing with other professional responsibilities (37). Addressing these barriers may require targeted interventions such as mentorship programs or dedicated time for research activities.

Most participants reported that the MPH program met or exceeded their expectations, particularly in terms of skill development and career preparation. However, unmet expectations were noted in areas such as practical training and course content. Participants suggested incorporating topics like epidemic outbreak management, digital health, policy analysis, and qualitative research which are the fields increasingly relevant in contemporary public health practice as indicated by Kickbusch et al. (39). Additionally, the discussion around assignment styles and teaching formats highlights the need for ongoing curriculum refinement. Active learning strategies, such as case-based learning and simulation exercises, have been shown to improve engagement and knowledge retention in public health education (34). Incorporating these elements more systematically could enhance the learning experience and better prepare graduates for the complexities of public health practice.

## 7 Strengths and limitations

One of this study's key strengths is its ability to capture the perspectives of graduates, who are best positioned to assess the program's impact on their career development and professional competencies. The inclusion of both structured surveys and qualitative interviews enhances the reliability of the findings by providing a more nuanced understanding of graduates' experiences. Additionally, the study offers valuable recommendations for improving the curriculum, program structure, and research engagement, making it directly relevant to public health educators and policymakers.

Despite these strengths, the study has certain limitations. The response rate for the survey was relatively low (25%), which may introduce response bias and limit the generalizability of the findings. The study relied on self-reported data of the questionnaires, which may be subject to recall bias or social desirability bias. The convenience sampling approach used for participant recruitment may have also contributed to selection bias, potentially over representing graduates with strong opinions about the program. Furthermore, as the study focused on a single MPH program, its findings may not be fully applicable to other public health programs in the region. Future research could benefit from a larger sample size, longitudinal tracking of graduates, and comparative analyses with similar programs in other institutions.

## 8 Recommendations and policy impact

To maximize the effectiveness of the MPH program and better support its graduates, several key improvements should be considered. One of the most pressing needs is the expansion of hands-on learning opportunities. Practical training remains a cornerstone of public health education, allowing students to bridge the gap between theory and real-world application. A comprehensive review of experiential learning programs demonstrates significant improvements in student satisfaction, problem-solving abilities, and communication skills (43). Moreover, establishing stronger partnerships with healthcare organizations, governmental agencies, and non-governmental organizations could create more opportunities for students to gain field experience is highly recommended. Regional networks like EMPHNET have demonstrated the value of collaborative approaches in strengthening public health systems and workforce development (44). Internship programs and community-based projects should be further integrated into the curriculum to ensure that graduates leave with not only theoretical knowledge but also the practical skills required to navigate complex public health challenges. Evidence-based frameworks for sustainable academic-industry partnerships have shown significant improvements in graduate work readiness and employer satisfaction (45).

In addition to strengthening practical training, there is a need for more structured career support. Many graduates felt that their degree was not sufficiently recognized by employers, limiting their job prospects after completing the program. To address this, the university could work more closely with employers to align the curriculum with workforce needs, ensuring that graduates are equipped with the skills most in demand in the public health sector. Lessons from comparative workforce development initiatives in the region, such as those implemented in Abu Dhabi, provide valuable insights for curriculum alignment with local market demands (46). Mentorship programs connecting students with experienced professionals could also play a crucial role in guiding career development, and structured mentorship initiatives at leading institutions have demonstrated success in supporting both career advancement and research productivity among public health graduates (47, 48). Furthermore, workshops focused on career preparation including resume writing, job search strategies, and interview techniques could be incorporated into the program to help students transition more smoothly into the workforce.

Another area that requires attention is research training and scholarly engagement. While students gained valuable research skills, many struggled to find the time and support necessary to publish their work. Addressing this challenge could involve creating mentorship programs where faculty members actively support students in the publication process. Additionally, providing dedicated research time within the program, along with access to funding opportunities, could help graduates contribute more actively to academic discourse in public health. Furthermore, flexibility in course delivery is another critical factor to consider. Given that a significant proportion of students are working professionals, maintaining flexible class schedules, including evening and weekend options, remain essential. At the same time, exploring a hybrid model that blends in-person and online learning could cater to diverse learning preferences without compromising engagement. The inclusion of interactive and discussion-based teaching strategies could also further enhance the learning experience.

Finally, continuous curriculum development is essential to ensure that the program remains responsive to the evolving landscape of public health. Participants suggested incorporating more content on contemporary public health issues such as digital health, epidemic outbreak management, and health policy analysis. Recent competency frameworks emphasize the importance of continuous curriculum updates to address evolving challenges in public health practice (10). By integrating these emerging topics, the program can better equip graduates to address the complex and rapidly changing challenges in public health practice. As the field of public health continues to evolve, academic institutions must remain agile and responsive to the needs of their students and the broader workforce. This is particularly relevant in the Eastern Mediterranean region, where professionalizing public health education has become a strategic priority (49). By strengthening practical training, fostering employer partnerships, enhancing research opportunities, and refining course delivery, the MPH program at UAEU can further its impact in preparing the next generation of public health professionals. The experiences and perspectives of graduates serve as a crucial guide in this process, offering valuable direction for the ongoing improvement and success of the program.

## 9 Conclusion

Despite many reported challenges, the overall sentiment among graduates was positive, with many acknowledging the MPH's role in shaping their academic and professional journeys. While the program has been instrumental in equipping students with critical knowledge and research skills, the findings highlight areas that need further refinement to enhance both the educational experience and career outcomes of graduates. Our results suggest that while the MPH program is already making a meaningful impact, there are opportunities for enhancement that could further strengthen its role in public health education and workforce development.

## Data Availability

The raw data supporting the conclusions of this article will be made available by the authors, without undue reservation.
