# Supplementary material for: Graduates feedback on the master of public health program in the United Arab Emirates: a mixed method study
Source: Front Public Health. 2026 Jan 12;13:1717098. doi: 10.3389/fpubh.2025.1717098 (PMC12832790; doi:10.3389/fpubh.2025.1717098)
Supplement: Supplementary file 1 [file Table_1.docx]

**Supplementary Materials**

**Table S1:** Summary and synthesis of the qualitative data (n=15)

| **Major Theme** | **Sub-theme** | **Participant ID & year** | **Representative Quote** |
| --- | --- | --- | --- |
| Theme 1: Program Structure | Mode of Delivery (Face-to-face preference) | M2_2019 | "I prefer the attendance in the class... it helps you absorb the course much better compared to online" |
|  | Mode of Delivery (Online flexibility benefit) | F3_2018 | "My main challenge was travelling to Al Ain because I'm Staying in Sharjah, this was one of the challenging, but... when Corona came It helped me that this online learning it was something that I benefited from" |
|  | Program Track: Preference for Thesis-based | F11_2014 | "Course based is really enjoyable, but what we learned during thesis... like structuring your thesis... There are certain skills which are very important for the future, especially those who would like to have their research to be written. So, uh, I'd like to see our MPH with thesis... dissertation, yes" |
|  | Time and Schedule: Part-time flexibility | F14_2015 | "For me, when I was in a master program, I was also like what I am now, a PhD student; I was part-time. I was studying and working. So, the four days per month for each course allowed me to manage it between work and university" |
| Theme 2: Experiences and Influence | Influence on Knowledge: Research Methods | F9_2017 | "During my master, I get a benefit and it to enrich my knowledge about the type of the designs of studies" |
|  | Influence on Knowledge: Writing Skills | F5_2018 | "My writing skills become better this because they always asking about assignments" |
|  | Influence on Knowledge: Perspective & Wellness | F3_2016 | "It gave me different perspective to view disease, illness and treatment and new concept and approach about wellness" |
|  | Influence on Career: Job Application | F1_2018 | "I got it because I'm working on public health institution, so I felt like now I have some concepts in my mind and some information related to our research that we are doing now" |
|  | Influence on Career: Career Advancement | M7_2017 | "I was working as an assistant in school health and university program, when I graduated, they directly put me in charge of the head of department" |
| Theme 3: Learning Experiences & Curriculum | Instructional Methods: Group Work & Discussion | F2_2018, M2_2019 (sequential) | "I like the diverse methods on work group, working as a group and the discussions and the assessments that we have to do at the end of the course. And of course, the lectures were important. The group discussions are important also" |
|  | Instructional Methods: Satisfied with Faculty | Multiple (not specified) | Highly satisfied with faculty expertise and flexibility; "Knowledgeable instructors" |
|  | Learning Format: Flexible Schedule Satisfaction | Multiple (not specified) | Many students reported satisfaction with program flexibility of course time and faculty understanding |
| Theme 4: Expectations | Met Expectations: Career Change Opportunity | F3_2018 | "I was planning to change my career and from the first tier and after finishing the first tier of master's degree I got an interview with the in the Dubai Health Authority" |
|  | Unmet Expectations: Academic Writing Skills | F4_2018 | "So, I think academic writing is definitely something that needs to be stressed on more because that's the only thing where if you can confidently say that oh I'm able to review papers and I'm able to, you know, let's say come up with, you know, just I have so and so publications under me" |
| Theme 5: Gaps and Challenges | Challenge: Time Pressure | F12_2012 | "To come up with presentations and of course it was challenging because we need to work on the presentations while attending the course" |
|  | Challenge: Assignment Timeline Stress | F3_2018 | "Usually there is a task that we have to do on the 4th day, and that was really very stressful because, uh, because of shortage of time" |
|  | Challenge: Online Learning Complexity | F3_2018 | "Well, I remember it was challenging the way that even long running through it was online and also it was in Group work" |
|  | Challenge: On-campus Preference | F6_2018 | "I would like to be on campus. I don't like the online, you know because before I join the program of master program I was searching for many online programs, and I really was so amazed with the syllabus and I was about to join and but after that I realized that on campus It's much better you will meet with the people you will have connection. It's really something different from online" |
|  | Gap: Missing Curriculum Content | F12_2012 | "I felt that genomics public health genomics. Wasn't there. Population health wasn't there, and the qualitative part wasn't there. You know you had. You had to teach me a lot in the qualitative so I think these are the things that need to be there. Social determinant, behavioural science. All these things I think can be added to the program" |
|  | Gap: Lack of University Connection | F11_2014 | "The gap between the courses it created some sort of not continuous and then the flow of the information or the flow of the practice was not continuous" |
| Theme 6: Suggestions & Recommendations | Suggestion: Practical & Research Component | F10_2018 | "Again, I wish there was like a practical side or thesis. if there are defence something around that. Something likes to publish" |
|  | Suggestion: Publication Requirement | M8_2010 | "I would highly recommend that everyone should end up with a paper published somewhere" |
|  | Suggestion: Internship Program | F4_2018 | "I personally feel like that would really add so much value to the whole program Like it would just. Just be like the cream, you know. Because all that it's kind of like you know how I did like I did five years of medical school and there was so much that I learned. But you know, when it comes to practical application you get so much from that one year of experience" |
|  | Suggestion: Training & Professional Development | F9_2017 | "To have internship, to have a training or to have like to at least to be invited to a conference It was all sort of searching" |
|  | Suggestion: Post-Graduate Resources | F4_2018 | "So perhaps maybe more assignments or more exercises, or you know, like even more further reading material. To keep me in touch because, see what happens is once you graduate from the program, and if you're not in the same field, then you know it's just you become rusty and you know you may you may forget, or you may not be very ...you may not pick up where you left off" |

Participant IDs: Format is [Gender_number][Year of graduation], F = Female, M = Male

Year indicates MPH graduation year (e.g., F3_2018 = Female graduate #3 who graduated in 2018)
